# The role of autophagy in odontogenesis, dental implant surgery, periapical and periodontal diseases

**DOI:** 10.1111/jcmm.18297

**Published:** 2024-04-13

**Authors:** Sevinç İnan, Emre Barış

**Affiliations:** ^1^ Department of Oral Pathology, Faculty of Dentistry Gazi University Ankara Turkey

**Keywords:** ATG, dentistry, inflammation, pathogenesis, tooth

## Abstract

Autophagy is a cellular process that is evolutionarily conserved, involving the sequestration of damaged organelles and proteins into autophagic vesicles, which subsequently fuse with lysosomes for degradation. Autophagy controls the development of many diseases by influencing apoptosis, inflammation, the immune response and different cellular processes. Autophagy plays a significant role in the aetiology of disorders associated with dentistry. Autophagy controls odontogenesis. Furthermore, it is implicated in the pathophysiology of pulpitis and periapical disorders. It enhances the survival, penetration and colonization of periodontal pathogenic bacteria into the host periodontal tissues and facilitates their escape from host defences. Autophagy plays a crucial role in mitigating exaggerated inflammatory reactions within the host's system during instances of infection and inflammation. Autophagy also plays a role in the relationship between periodontal disease and systemic diseases. Autophagy promotes wound healing and may enhance implant osseointegration. This study reviews autophagy's dento‐alveolar effects, focusing on its role in odontogenesis, periapical diseases, periodontal diseases and dental implant surgery, providing valuable insights for dentists on tooth development and dental applications. A thorough examination of autophagy has the potential to discover novel and efficacious treatment targets within the field of dentistry.

## 1 INTRODUCTION

The initial scientific characterization of autophagy, derived from the Greek term ‘self‐eating’, pertained to the transportation of cytoplasmic cargo to the lysosome for destruction. Chaperone‐mediated autophagy (CMA), microautophagy and macroautophagy represent different types of autophagy, each characterized by unique mechanisms for delivering cargo to the lysosome.^1^ Microautophagy is a non‐selective form of lysosomal degradation in which cytoplasmic cargo is directly engulfed by autophagic tubing, which also causes cytoplasmic lumen entry and vesicle breakage. Autophagosomes are not used as transport intermediaries in microautophagy. Instead, autophagic cargo is engulfed and taken up directly by an endosome or lysosome.^2^ The process of CMA involves a series of steps aimed at specifically targeting and degrading soluble intracellular proteins. The process through which CMA transports proteins bearing the KFERQ motif into the lysosome involves the identification of these proteins by cytosolic HSPA8, a member of the heat shock protein family A (Hsp70). LAMP‐2A (lysosomal‐associated membrane protein 2A) multimerization is required for translocation into the lysosome.^3^ Macroautophagy is a catabolic process in eukaryotic cells that utilizes autophagosomes, which are double‐layered membrane structures. These autophagosomes fuse with lysosomes after maturation, leading to the destruction of vesicular contents like unorganized cytoplasm, dysfunctional organelles and microbial invaders through lysosomal hydrolysis. Autophagy, a cellular process, is regulated by autophagy‐related genes (ATG) that were initially discovered through genetic screens conducted in yeast.^4^ At present, the pathway that has been subject to the most comprehensive research is macroautophagy, which will be subsequently referred to as autophagy.

Autophagy may be additionally stimulated in the presence of stressors, such as lack of nutrients or energy, with the purpose of decomposing cytoplasmic components into metabolites that can be utilized for biosynthesis or energy generation, so enabling cellular survival.^1^ In typical developmental circumstances, autophagy selectively eliminates impaired or surplus organelles in order to facilitate cellular upkeep. Therefore, it may be said that macroautophagy primarily functions as a system for cellular protection. However, it is important to note that an excessive level of self‐degradation can have negative consequences. Autophagic dysfunction is related to a number of oral and systemic diseases, such as diabetes, heart disease, neurodegenerative diseases, oral cancer, periodontal and periapical disorders.^5^, ^6^, ^7^

This review was conducted by searching the medical literature from the PubMed® scientific database from 01 January 2010 to the present day. Keywords used in the search were autophagy and odontogenesis/tooth formation for odontogenesis chapter, autophagy and periapical disease/lesions/granuloma for periapical disease chapter, autophagy and periodontal disease/periodontitis/alveolar bone loss/periodontal medicine for periodontal disease chapter, and autophagy and dental implant/osseointegration for dental implant surgery chapter. From the results, the most relevant and recent studies on autophagy and the specific areas examined in this review were included. Studies that were not in English and for which the full text was not available were excluded.

The aim of this study is to present a comprehensive and up‐to‐date literature review on the dento‐alveolar effects of autophagy, which is known to be involved in the pathogenesis of many diseases, with a particular focus on its role in odontogenesis, periapical diseases, periodontal diseases and dental implant surgery. This review will be an important resource especially for dentists in terms of revealing the role of autophagy in tooth development and specific dental applications.

## 2 MECHANISMS OF AUTOPHAGY

**FIGURE 1:**
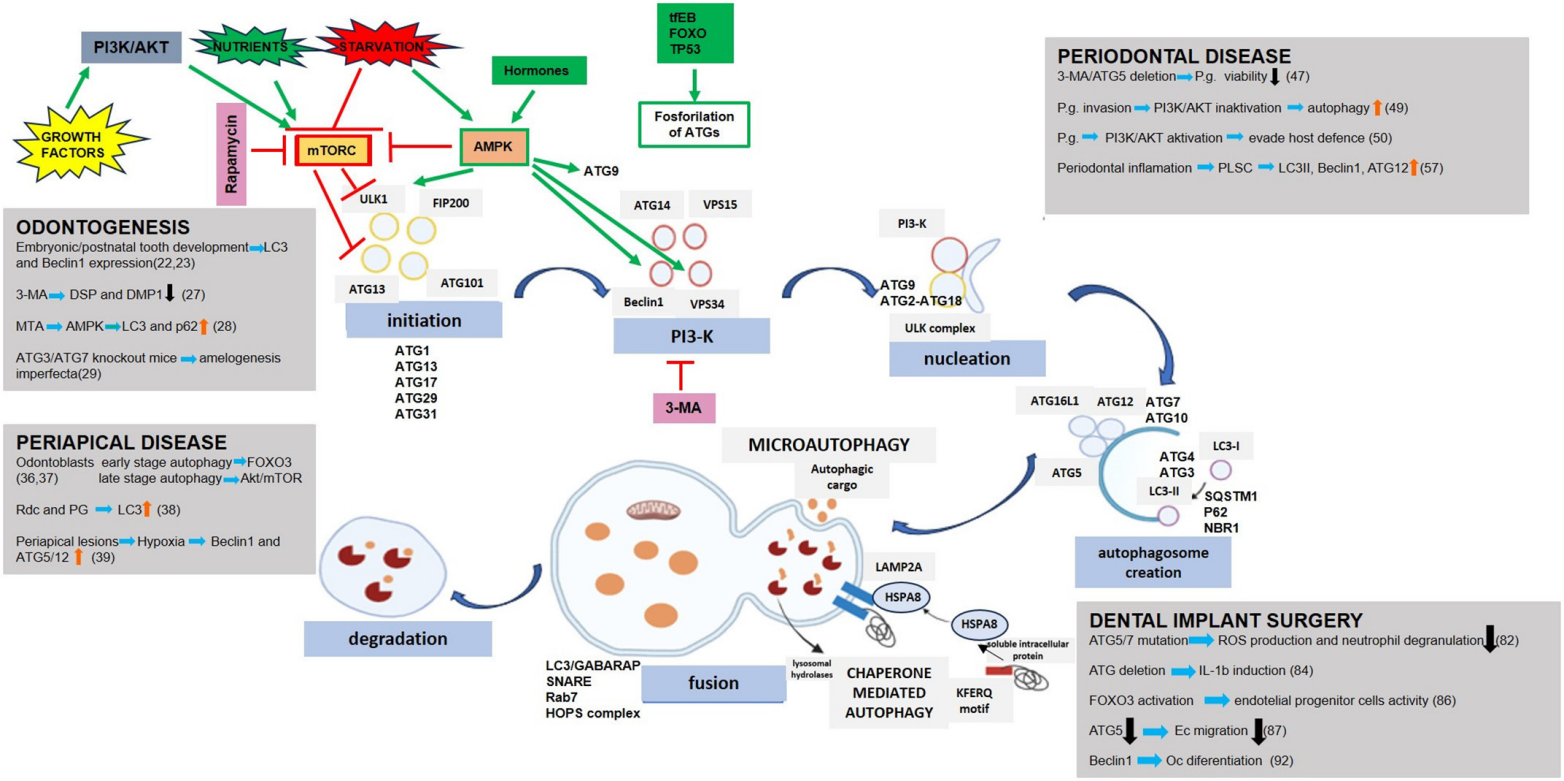
Autophagy mechanism is mainly driven by ATGs. Degradation occurs through membrane invagination in microautophagy and through LAMP2A in chaperone‐mediated autophagy. The main regulators of autophagy are mTORC and AMPK, but hormones and transcription factors are also involved in autophagy regulation. Autophagy has important implications in tooth development, dental implant surgery and the development of periapical and periodontal diseases. (Green arrow: activation; red arrow: inhibition; orange arrow: increase; black arrow: decrease).

Autophagy is a cellular process that involves the participation of a set of 16–20 core conserved ATG. Figure 1 illustrates the mechanisms of autophagy in the dentoalveolar region. As of the present time, a total of 41 ATG have been identified. 18 ATG genes, ranging from ATG‐1 to ATG‐10, ATG‐12 to ATG‐14, ATG‐16 to ATG‐18, ATG‐29 and ATG‐31, encode proteins necessary for autophagosome formation. Autophagy involves initiation, cargo packing, elongation of the phagophore, autophagosome creation, lysosomal fusion and disintegration.^5^ The ULK1 serine threonine kinase complex, which consists of ULK1, FIP200, ATG‐13 and ATG‐101, serves as the primary component in the initiation of autophagy by phosphorylating a number of downstream components. The class III phosphatidylinositol 3‐kinase (PI3K‐3) complex plays a crucial role in the initiation of phagophore production.^8^ When PI3K‐3 is activated, it generates phosphatidylinositol‐3‐phosphate (PI3‐P), which plays a crucial role in initiating the phagophore area by attracting PI3‐P effector proteins such as WIPI2 and ZFYVE1/DFCP1.^9^ There are two distinct complexes that are involved in the production of PI3‐P for separate cellular processes. The first complex, consisting of Beclin 1, VPS34, VPS15 and ATG‐14, is responsible for autophagosome nucleation. The second complex, comprising Beclin 1, VPS34, VPS15 and UVRAG, is involved in the maturation of endolysosomes and autophagolysosomes.^8^ After nucleation, the phagophore grows due to the activity of two ubiquitin‐like systems. ATG12 is linked to ATG5 by the E1‐like enzyme ATG7 and the E2‐like enzyme ATG10 in the initial system and subsequently combines with ATG16L1 to create a complex. In the second system, Atg8‐family proteins such as LC3 and GABARAP subfamilies are cleaved by ATG4 and then attached to phosphatidylethanolamine (PE) through the enzymes ATG7, ATG3 and the ATG12–ATG5‐ATG16L1 complex.^10^ ATG16L1 directly interacts with WIPI2, facilitating the conjugation process at the phagophore membrane.^11^ The phagophore will enlarge into a cup‐shaped structure that isolates cytoplasmic components, and the phagophore's closing forms the autophagosome. The outer membrane of the autophagosome merges with an endosome and then with the lysosome. Inside the lysosome, the inner membrane and its contents are broken down and reused after being released back into the cytosol through permease.^12^

## 3 REGULATION OF AUTOPHAGY

Autophagy helps cells cope with a variety of extracellular and intracellular stresses, such as endoplasmic reticulum (ER) stress, hypoxia and the condition of nutritional deprivation, as well as endocrine factors.^1^ The mTORC (mechanistic target of rapamycin) plays a crucial role in inhibiting autophagy, a cellular process involved in nutrition sensing. mTORC is stimulated by the PI3‐K and AKT/protein kinase B (PKB) signalling pathways in the presence of nutrients or in reaction to growth factors. Following activation, mTORC partially suppresses autophagy by phosphorylating ULK1 and ATG‐13.^13^ The activation of adenosine monophosphate activated protein kinase (AMPK) occurs as a result of adenosine triphosphate (ATP) depletion. This activation leads to the phosphorylation of various proteins, hence stimulating catabolic pathways and inhibiting anabolic pathways. This process effectively restricts the utilization of adenosine triphosphate (ATP) and facilitates the production of fresh ATP through the degradation of metabolic by‐products.^14^ The activation of autophagy by AMPK occurs via the inhibition of mTORC and the direct phosphorylation of various ATG proteins, such as ULK1, ATG‐9A, Beclin 1 and VPS34.^15^ Autophagy is also regulated at the transcriptional level by factors such as transcription factor EB (tfEB) FOXO, TP53.^16^ Lysine acetylation or deacetylation of ATG proteins has an important role in autophagic control. Sirtuin 1 is responsible for the deacetylation of a set of ATG, including ATG‐5, ATG‐7, ATG‐12, Beclin 1, VPS34 and LC3.^17^, ^18^ Exposure to ER stress prompts cells to enhance protein breakdown, and autophagy aids in removing accumulated proteins. Extended endoplasmic reticulum stress results in autophagy‐induced cell death.^19^ Many hormones control autophagy and thus metabolism by regulating AMPK activity.^20^ Rapamycin, BH3 mimetics and lithium can stimulate autophagy by inhibiting negative autophagic regulators like mTORC. Autophagy can be hindered in the initial phases by blocking the PI3‐K complex with 3‐methyladenine (3‐MA).^5^

## 4 AUTOPHAGY AND ODONTOGENESIS

Teeth form through intricate epithelial–mesenchymal interactions. Teeth initiation, morphogenesis, odontoblast and ameloblast differentiation, dentin and enamel creation depend on these interactions.^21^ The process of autophagy is of crucial significance in the development of teeth. LC3 and Beclin 1 exhibit constant expression across all phases of embryonic and postnatal tooth development.^22^, ^23^ Autophagy is increased in the cervical loop and primary enamel knot of the dental papilla and enamel organ during embryonic development. This shows that autophagy regulates tooth morphogenesis and root development. Postnatal tooth germ cells such dental epithelial cells, odontoblasts, dental follicle cells and Hertwig's epithelial root sheath cells do autophagy. This shows that autophagy differentiates dental precursor cells, possibly promoting enamel and dentin formation.^22^

Apoptotic cells that are located in the primary enamel knot, and stellate reticulum have autophagic vacuoles in their cytoplasm. The coexistence of autophagy and apoptosis between the primary enamel knot and the stellate reticulum indicates that autophagy plays a regulatory role in the dynamic cellular processes occurring in signalling centres involved in tooth formation.^24^ Autophagy is observed in non‐apoptotic cells within the dental germ, and it has been proposed as an adaptive mechanism to protect ameloblasts against cell stress and oxidative harm caused by fluoride exposure.^25^

During the postnatal phase of tooth development, odontoblasts undergo a process known as autophagy.^23^ The suppression of autophagy enhances the process of odontoblast differentiation triggered by fibroblast growth factor 2 (FGF‐2), which is regulated by core binding factor 1 (Cbf‐1) through the Wnt I signalling pathway. There is a proposition that the induction of FGF‐2 leads to the transcriptional promotion of Cbf1, which subsequently enhances the expression of dentin sialophosphoprotein. This, in turn, facilitates the differentiating of odontoblasts by inhibiting the process of autophagy.^26^ In a separate investigation, it was discovered that mineral trioxide aggregate facilitated the process of odontoblastic differentiation in human dental pulp cells when autophagy was induced. Conversely, the inhibition of autophagy by the use of 3‐MA resulted in a notable decrease in the expression levels of dentin sialophosphoprotein and dentin matrix protein 1.^27^ The administration of mineral trioxide aggregate following cavity preparation in the molars of rats was seen to induce an upregulation of dentin matrix protein 1 and dentin sialophosphoprotein, as well as an increase in the levels of LC3II and p62, along with an enhancement in the phosphorylation of AMPK.^28^ A study in autophagy‐impaired mice in epitel‐derived tissues shows that autophagy plays an important role in ameloblast differentiation.^29^

Autophagy has the potential to induce the migration of dental pulp stem cells and facilitate the process of pulp regeneration. Autophagy has the potential to induce the migratory capacity of dental pulp stem cells, hence promoting the process of pulp regeneration. During the process of SDF‐1α‐mediated pulp revascularization in pulpectomized canine teeth that have achieved complete apical closure, there was an observed increase in the levels of ATG‐5 and LC3.^30^ The process of autophagy plays a crucial role in facilitating the osteogenic differentiation of dental pulp stem cells. The activation of AMPK signalling pathway triggers the process of autophagy, which is essential for the initial stages of osteogenic differentiation in dental pulp stem cells (DPSc).^31^ Previous studies have demonstrated the involvement of autophagy in the sodium fluoride‐induced osteogenic and odontogenic differentiation process of stem cells derived from the apical papilla and that interleukin‐37 (IL‐37) has the ability to enhance the osteogenic and odontogenic differentiation process in DPSc by promoting autophagy.^32^, ^33^ Autophagy has been demonstrated during the embryonic and postnatal stages of tooth development and is involved in the dentin, enamel, root formation and differentiation of dental pulp cells. Further studies focusing on the role of autophagy during the stages of tooth development may provide an effective mechanism for the prevention of tooth development disorders.

## 5 AUTOPHAGY AND PERIAPICAL DISEASE

Emerging evidence suggests a role for autophagy in the pathology of pulpitis. The occurrence of autophagy and mitophagy is notably heightened in dental pulp samples affected by caries and inflammation.^34^ Autophagy consistently manifests in the course of irreversible pulpitis in a rat model.^35^ In lipopolysaccharide (LPS)‐stimulated odontoblasts, autophagy has been shown to have a dual time‐dependent role. In the first phase of pulpitis, autophagy plays a crucial role in preserving cellular viability, however in the advanced stage of pulpitis, sustained autophagy contributes to the induction of apoptosis.^36^ The activation of early‐stage autophagy in odontoblasts is facilitated by FOXO3a, while the progressive activation of AKT/mTORC/survivin is responsible for late‐stage autophagy.^36^, ^37^

Periapical lesions are necrosis of dental pulp tissue related to an inflammatory response exacerbated by infections, also referred to as periapical granulomas (PG). Autophagy is a commonly observed phenomenon in both PG and radicular cysts (RdC), primarily occurring within inflammatory cells. All samples of RdC and PG show typical autophagosomes and strong LC3 staining.^38^ Hypoxia is present at the centre of periapical lesions due to ischemia and nutritional deficiency. The levels of hypoxia‐related and autophagy‐related proteins (AMPK, HIF‐1a, BNIP3, Beclin 1 and ATG‐5,12) were observed to be elevated in periapical lesions in comparison to normal pulp tissue.^39^ The present investigation examined the effects of hypoxia on mitochondrial function and mitophagy in osteoblasts within a rat model of generated apical periodontitis. The induction of hypoxia in osteoblasts also resulted in the activation of apoptosis, and the suppression of mitophagy decreased the apoptotic activity that was boosted by hypoxia.^40^

The regulation of apoptosis and autophagy is crucial in maintaining the equilibrium between cell survival and cell death in periapical lesions.^38^, ^41^ The process of autophagy exhibits a colocalization phenomenon with apoptotic signalling in PGs and RdC, predominantly observed in foamy macrophages.^38^ Apoptosis or a caspase‐independent form of cell death can both be promoted by autophagy activation.^38^, ^42^ The presence of mutations in ATG and the impairment of autophagic activity have been found to heighten susceptibility to intracellular pathogen infections.^43^ The rat periapical lesion model has demonstrated a negative association between autophagy and apoptosis. The periapical lesions that were treated with simvastatin exhibited reduced bone degradation, increased expression of Beclin 1 and inhibited apoptosis of osteoblasts.^41^ Autophagy has the potential to exert a negative regulatory influence on apoptosis, hence contributing to its cytoprotective function.^44^ Nevertheless, existing evidence indicates that an overabundance of autophagy leads to cellular demise, while the suppression of autophagy seems to induce macrophage cell death that is independent of caspase activity.^42^ Therefore, further investigation is necessary to elucidate the interplay between autophagy and apoptosis in periapical lesions.

## 6 AUTOPHAGY AND PERIODONTAL DISEASE

Periodontal disease (PD) is a persistent inflammatory ailment that impacts the alveolar bone, gingiva, and other structures responsible for tooth support. When the interaction between pathogens and the human immune system is out of balance, PD occurs. PD is characterized by the presence of Gram‐negative periodontal bacteria, including Porphyromonas gingivalis (P.g.) and Aggregatibacter actinomycetemcomitans (A.a.), which constitute the predominant subgingival plaque. P.g. is a notable opportunistic pathogen associated with PD that has been the subject of substantial research. Autophagy is essential for supporting the survival of periodontal pathogenic bacteria. P.g. has the ability to employ autophagy as a mechanism to circumvent the host's immune systems, hence enhancing its ability to penetrate and colonize the periodontal tissues of the host organism.^45^ It has been shown that P.g. and its LPS promote autophagic activity.^46^ Based on the findings of a study, the viability of P.g. inside gingival epithelial cells (GEC) was observed to undergo a notable decrease when the process of autophagy was impeded through the use of 3‐MA or deletion of ATG‐5.^47^ P.g. localizes in autophagosomes and interferes with the development of autolysosomes, which causes an accumulation of autophagosomes that provide nutrition for the survival of the bacteria.^46^ The PI3K/PKB (AKT)/mTOR signalling pathway plays a crucial role in regulating autophagy. Inactivation of this pathway leads to autophagy following invasion by P.g.^48^ Inducing autophagy in GEC creates a beneficial environment for their survival and avoidance of immunological responses.^49^ In addition, a recent study has discovered that P.g. employs a mechanism to influence the autophagic process by activating the AKT/mTORC signalling axis. This enables P.g. to evade immune surveillance and persist within dendritic cells.^50^ Additionally, the antibacterial process was increased by the stimulation of autophagy in immune cells.^51^ Further investigation is warranted to elucidate the intricate interplay connecting autophagy and periodontal pathogens in the aetiology of PD.

Autophagy functions as a component of the innate immune system's effector mechanism, successfully restricting the infiltration of pathogens.^52^ The autophagic flux is increased by periodontal bacteria in both the periodontal tissues and immune cells. Beclin 1 and LC3‐II expression and ATG‐5, ATG‐12 conjugation increased in THP‐1‐derived macrophages after P.g. invasion. In addition, autophagy suppression decreased P. g. viability.^53^ Upon infection of THP‐1 cells, the induction of an autophagic response was seen, resulting in the inhibition of intracellular survival of A.a.^51^

Autophagy and gingival cell release of nuclear high mobility group box 1 are linked. In addition, it attracts polymorphonuclear leukocytes and acts as an inflammatory and immunological stimulant in PD.^54^ According to research findings, individuals diagnosed with periodontitis have increased concentrations of oxidative damage products in their serum, plasma, and peripheral blood mononuclear cells, along with reduced antioxidant capacity.^55^ Oxidative stress primarily leads to elevated levels of reactive oxygen species (ROS), consequently causing damage to periodontal tissues.^56^ Mitochondrial ROS at heightened concentrations have the potential to induce autophagy in individuals diagnosed with periodontitis.^55^

Inflammation induces autophagy, which protects against apoptosis. Inflammation with TNF‐α and IL‐1 led to increased expression of LC3‐II, Beclin 1 and ATG‐12 in periodontal ligament stem cells (PLSC).^57^ The application of TNF resulted in a significant increase in autophagy levels in PLSC, while concurrently suppressing apoptosis.^58^ Furthermore, the suppression of autophagy through the use of 3‐MA resulted in an elevation of apoptosis in gingival fibroblasts (Fb).^56^ Therefore, autophagy may potentially provide a protective mechanism in the presence of inflammation.

Anabolic and catabolic processes are regulated by cytokines, ensuring tissue homeostasis. TGF‐β is thought to have a crucial role in regulating structural cell development and mobility, particularly in inflammation and immune response regulation.^59^ TGF‐β1 is important for wound healing, contributing to tissue fibrosis, myofibroblast (MFb) development and perhaps increasing extracellular matrix (ECM) in pathological situations.^60^ It suppresses pro‐inflammatory cytokines such IL‐1, TNF‐α and metalloproteinases (MMP) and has powerful immunosuppressive effects.^61^ In a study, TGF‐ β1 levels were found to be high in patients with chronic periodontitis.^59^ Knocking down Periodontal ligament‐associated protein‐1 has been shown to hinder osteoclast development and decrease alveolar bone resorption via the TGF‐β1/Smad1 signalling pathway.^62^ This results suggest that high TGF‐β1 may be a protective factor for periodontitis, given that it accelerates connective tissue remodelling and cell differentiation. Various investigations across different organs have demonstrated a direct relationship between activated autophagy and MFb development in tissue regeneration.^63^ The activation dynamics of human Fbs from healthy oral mucosa or gingiva indicated that autophagy directly affects MFb growth. Autophagy activation and enhanced a‐SMA and collagen deposition in oral mucosa Fbs after surgery. Gingiva Fbs that could not express autophagy did not differentiate into MFbs.^64^ TGF‐β controls autophagy and MFb differentiation, but the specific mechanisms of regulation and interaction between the two pathways are not well understood. Several studies have shown that TGF‐β stimulates autophagy in various Fb systems, whereas others have proposed the contrary.^63^ Research has demonstrated that inhibiting autophagy effectively stops TGF‐β from activating MFbs in gingival Fb cells.^64^ Although these controversial results clearly show a relationship between autophagy and the TGF‐β pathway, further research is needed to fully elucidate this relationship in periodontal disease.

PD is closely associated with an increased risk of developing many human diseases, including cardiovascular diseases (CVD), neuroinflammatory illnesses and diabetes.^65^ Large cohort clinical trials like the Periodontitis and Its Relation to Coronary Artery Disease (PAROKRANK) study have confirmed the link between periodontitis, heart failure and CVD. This study found that periodontitis patients had greater cardiovascular disease biomarkers and a higher risk of early myocardial infarction than matched controls.^66^ Periodontal inflammation has been shown to potentially elevate the risk of cardiovascular disease by triggering an elevation in inflammatory markers such hs‐CRP and NT‐proBNP when compared to persons without PD. Moreover, the greater the degree of periodontal destruction, the higher the levels of NT‐proBNP in serum.^67^ Periodontal health in individuals is associated with adverse changes in CRP and NT‐proBNP levels, as well as CVD.^68^ Studies have also connected periodontal therapy with NT‐proBNP levels. Patients with chronic periodontitis who had nonsurgical periodontal therapy showed a notable reduction in both gingival crevicular fluid and serum NT‐proBNP levels.^69^ NT‐proBNP levels were elevated in the periodontitis group compared with the control group and decreased after periodontal flap surgery.^70^ In a separate research, full‐mouth scaling and root planning was more efficient than regular oral care in decreasing clinical factors and NT‐proBNP levels. Participants with high NT‐proBNP levels initially experienced greater clinical improvements following periodontal therapy during a 6‐month period.^71^ Treatment with tifanoside in rats reduced the LC3‐II/LC3‐I ratio and elevated P62 protein expression. Additionally, it enhanced the p‐AKT/AKT and p‐mTOR/mTOR ratios considerably. There was a notable rise in the expression of p62 protein, as well as in the ratios of p‐AKT/AKT and p‐mTOR/mTOR. Serum levels of NT‐proBNP, IL‐6, TNF‐α, MMP‐2 and MMP‐9 were observed to have decreased.^72^ In a study conducted in cardiac patients, an association between autophagy and serum levels of NT‐proBNP, CRP, IL‐6, TNF‐α and TGF‐β1 was reported.^73^ It is noteworthy that mediators such as NT‐proBNP, which increase in CVD, decrease with periodontal treatment and autophagy inhibition in CVD patients, although studies are insufficient. Further studies on this subject will help to better understand the relationship between PD and CVD.

Alzheimer's disease (AD) is the most common neurodegeneration in seniors. Key features of AD include amyloid beta (Aβ) accumulation and excessive tau phosphorylation in the brain. Positron emission tomography has confirmed the accumulation of Aβ in specific brain regions of Parkinson's disease patients compared with controls. This is supported by the analysis of Aβ peptide levels in the blood, which showed different expressions in individuals with periodontal issues Recent research reveals that autophagy may treat neurodegeneration. A study demonstrated that Aβ generation was reduced by the regulation of autophagy reliant on 3‐MA in an in vitro setting.^74^ Tau post‐translational modification can hinder axoplasmic flow through autophagy, particularly affecting dynein–dynactin motors in vitro.^75^

Periodontitis associated with type 2 diabetes mellitus is a common disease with high prevalence, persistent infection and complicated symptoms. Diabetic rats have shown an intense inflammatory response and reduced autophagy compared with non‐diabetic rats.^76^ Argpyrimidine, one of the main products of diabetes, has been implicated in autophagy of PDLCs via PI3K/AKT/mTOR.^77^ High glucose hindered the activity of PLSC in diabetic rat models, but the modulation of autophagy helped maintain cell function. Modulating autophagy somewhat counteracted the adverse impact of high glucose circumstances on PLSC.^78^ Diabetic rats showed alterations in LC3, Bax and Bcl‐2 in periodontal tissue, suggesting the involvement of autophagy and apoptosis in the injury to periodontal tissue in diabetic rat.^79^ A study determined that apoptosis and autophagy are linked to the generation of ROS. Furthermore, autophagy induced by AGEs played a role in decreasing ROS generation and safeguarding against AGEs‐triggered apoptosis in PDLC.^80^ These results suggest that autophagy is involved in the process of diabetes and periodontal tissue damage.

## 7 AUTOPHAGY AND DENTAL IMPLANT SURGERY

Dental implants are biocompatible, synthetic materials surgically implanted into the maxilla and mandible. Their objective is to treat tooth loss and restore missing orofacial components. Neoplasia, trauma and congenital anomalies may have destroyed these components. After implant surgery, soft tissue wound healing and implant‐bone integration must be prioritized. The haemostasis and inflammation, proliferative and remodelling phases of soft tissue wound healing are carefully coordinated. Neutrophils play a crucial role in the inflammatory phase of wound healing subsequent to oral surgery, as they infiltrate the wound site to engage in phagocytosis and eradicate any present microbial contaminants.^81^ A research investigation conducted on mice exhibiting mutations in the ATG‐5/7 showed an intense reduction in the levels of neutrophil degranulation and ROS generation.^82^ Autophagy is strongly correlated with the specific physiological functions carried out by neutrophils. Oral tissue macrophages, originating from circulating monocytes, display migratory activity towards the injury site and are crucial for immune regulation. They also secrete growth factors that are important for stimulating the growth and movement of connective tissue cells in the periodontium.^81^ A study indicates that the ERK‐Beclin‐1‐autophagy axis may have a significant impact on the control of alternate macrophage polarization caused by nanotopography.^83^ Another study has found that inhibition of autophagy, pharmacological intervention or deletion of the ATG resulted in the induction of IL‐1b secretion in macrophages.^84^ The findings showed that autophagy restricts macrophage inflammation. Restriction of macrophage inflammatory response may help wound healing after implant surgery and implant integration with bone tissue. Endothelial cells (Ec) have an important role in angiogenesis in wound healing. The phenomenon of hypoxia‐induced autophagy can be regarded as a protective molecular mechanism employed by Ec and plays a significant role in facilitating angiogenesis, a crucial physiological process involved in the wound healing response following implant surgery.^81^ Stimulating autophagy in Ec promotes resistance to apoptosis, hence ensuring cell survival, and also provides protection against oxidative damage.^85^ A study shown that the activation of autophagy by FOXO3 enhanced the activities of endothelial progenitor cells.^86^ The inhibition of autophagy by the suppression of ATG‐5 expression or the use of autophagy inhibitors has been demonstrated to downregulate autophagy and thus hinder the migratory and tube‐forming capabilities of Ec.^87^ Further investigation of Ec autophagy may prove to be a helpful factor in achieving promising results in terms of successful healing outcomes in oral implant surgery. The presence of Fb‐rich barrier in close proximity to the outer layer of the implant is crucial for the initiation and maintenance of an effective seal against external elements. Fb's capacity to move and differentiate is the key factor in the formation of a barrier rich in Fb. The involvement of autophagy in the modulation of Fb differentiation has been elucidated.^81^ The study investigated the spatiotemporal alterations in LC3‐positive spots inside Fbs and MFbs in a rat wound healing model. The results demonstrated a notable increase in the number of LC3‐positive spots throughout the last stages of the proliferative phase.^88^ Subsequent research has shown that autophagy does not play a role in gingival wound healing and is linked to decreased differentiation of MFbs.^64^ Autophagy's role in wound healing can have a dual impact and result in different clinical results, depending on the precise type of cell involved. The process of epithelial regeneration involves crucial stages, including the process of keratinocyte (Kc) growth and migration from the outside edges of the wound towards its central region.^89^ The autophagic pathway has been shown to drive the proliferation and migration of Kc through CCL2, as well as initiating Fb activation.^90^ A hypoxic microenvironment has been shown in early wound healing. Hypoxia increases ROS, which activates BNIP3‐mediated autophagy. Overexpression of autophagy activated p38 and JNK‐MAPK signalling pathways to drive Kc migration.^91^ The promotion of Kc migration and proliferation by autophagy confers a significant beneficial role on this molecular pathway during the wound healing phase of dental implant surgery.

The pivotal factor for achieving effective implant osseointegration and mitigating the occurrence of implant rejection is the maintenance of a balance between osteoblasts (Ob) and osteoclasts (Oc). The role of Beclin 1 in the differentiation of Oc has been revealed.^92^ The proteins ATG‐5, ATG‐7, ATG‐4B and LC3 have been found to exhibit a function unrelated to autophagy in the processes of frilly border formation and bone resorption in Oc.^93^ Furthermore, it has been shown that the functions of Oc require conversion from LC3‐I to LC3‐II but do not increase autophagic flux.^92^

Autophagy has been demonstrated to have a significant role in the regulation of osseointegration, particularly in the context of implants within the osteoimmune milieu.^94^ In order for osteoblasts to direct mineralization during the formation of bones, autophagy is essential. Autophagy can be conceptualized as a mechanism that facilitates the transportation of mineralization‐related components, which are enclosed within stromal vesicles, to the extracellular matrix.^94^ Researchers have conducted experiments utilizing Ob cell lines to provide evidence that the process of autophagic flux is enhanced during the differentiation of Ob cells and the following mineralization process.^95^ A study conducted on rats demonstrated that simvastatin has the potential to augment the process of osteoblastic development in bone marrow‐derived mesenchymal stem cells by promoting autophagy and reducing osteoclast activity.^96^ In addition, studies on different implant surface areas have also shown that autophagy plays important roles in osteoblast differentiation and survival of osteoblasts and is effective in osseointegration.^97^, ^98^

This finding underscores the significant contribution of autophagy to these two critical processes, which are essential for the healing of bone lesions and the successful integration of dental implants with the surrounding bone tissue. However, most of the limited research on autophagy's roles in dental implant osseointegration is in vitro. Dental implants are strategically positioned within the oral cavity, which presents a complex microenvironment. Further investigation is required to have a comprehensive understanding of the intricate correlation between autophagy and the in vivo process of implant osseointegration.

## 8 CONCLUSION

Researchers have made big steps forward in understanding autophagy, which is helping us learn more about oral conditions like tooth development, periapical diseases, periodontal diseases and dental implant surgery. Autophagy helps dental precursor cells differentiate and may play a role in the creation of enamel and dentin, which controls tooth shape and root growth. Autophagy may also contribute to the pathogenesis of periapical diseases and, on the other hand, limit inflammation. Autophagy has important effects in promoting the survival of periodontal pathogenic bacteria, in the immune response. Autophagy may play a protective role in PD via TGF‐β. Autophagy may emerge as an important mechanism in the relationship between PD and other systemic diseases. Autophagy promotes wound healing. The pivotal significance of autophagy in osteogenesis is evidenced by its participation in the mechanisms of osteoblast differentiation and maturation. Nevertheless, the specific processes that underlie the involvement of autophagy in the onset and advancement of oral illnesses remain mainly unexplored. Therefore, additional research is required to gain a more comprehensive understanding of the interplay between autophagy, apoptosis, the immunological response and other cellular mechanisms in the context of oral illnesses. This review provides comprehensive and up‐to‐date information on autophagy and diseases in the dentoalveolar region and suggests that a comprehensive investigation of autophagy has the potential to reveal new and effective therapeutic targets in dentistry.

## AUTHOR CONTRIBUTIONS

**Sevinç İnan:** Conceptualization (equal); data curation (equal); formal analysis (equal); methodology (equal); visualization (supporting); writing – original draft (lead); writing – review and editing (lead). **Emre Barış:** Conceptualization (equal); data curation (equal); formal analysis (equal); methodology (equal); visualization (equal); writing – original draft (equal); writing – review and editing (equal).

## CONFLICT OF INTEREST STATEMENT

The authors confirm that there are no conflicts of interest.

## Data Availability

Data is openly available in a public repository that issues datasets with DOIs.
